# Molecular aspects of bacterial nanocellulose biosynthesis

**DOI:** 10.1111/1751-7915.13386

**Published:** 2019-03-18

**Authors:** Paulina Jacek, Fernando Dourado, Miguel Gama, Stanisław Bielecki

**Affiliations:** ^1^ Institute of Technical Biochemistry Lodz University of Technology 4/10 Stefanowskiego Str 90‐924 Lodz Poland; ^2^ Centre of Biological Engineering University of Minho Campus de Gualtar 4710‐057 Braga Portugal

## Abstract

Bacterial nanocellulose (BNC) produced by aerobic bacteria is a biopolymer with sophisticated technical properties. Although the potential for economically relevant applications is huge, the cost of BNC still limits its application to a few biomedical devices and the edible product Nata de Coco, made available by traditional fermentation methods in Asian countries. Thus, a wider economic relevance of BNC is still dependent on breakthrough developments on the production technology. On the other hand, the development of modified strains able to overproduce BNC with new properties – e.g. porosity, density of fibres crosslinking, mechanical properties, etc. – will certainly allow to overcome investment and cost production issues and enlarge the scope of BNC applications. This review discusses current knowledge about the molecular basis of BNC biosynthesis, its regulations and, finally, presents a perspective on the genetic modification of BNC producers made possible by the new tools available for genetic engineering.

## Introduction

Bacterial nanocellulose (BNC) is a linear polysaccharide composed of β‐d‐glucopyranose monomers linked by β‐1,4‐glycosidic linkages. The repeating unit is the disaccharide cellobiose (McNamara *et al*., 2015). Bacterial nanocellulose was first described in 1886 by A. J. Brown, who observed the production of cellulose by *Acetobacter xylinum* cells in the presence of oxygen and glucose (Brown, 1886). Meanwhile, classification of acetic acid bacteria has changed, *Acetobacter* being reclassified as *Gluconacetobacter*, which has recently further moved to a new type of *Komagataeibacter*. The current generic name as proposed in 2012 by Yuzo Yamada (Yamada *et al*., 2012) comes from the name of the Japanese microbiologist Kazuo Komagata.

Among the many BNC producers, we can distinguish nitrogen‐binding bacteria (*Rhizobium leguminosarum*), plant pathogens (*Dickeya dadantii*), *Agrobacterium tumefaciens*,* Escherichia coli*,* Salmonella enterica*,* Pseudomonas putida* and bacteria of the genus *Komagataeibacter*, currently regarded as one of the most efficient producers of this exopolysaccharide (Römling and Galperin, 2015). Current literature indicates a particular interest in bacterial strains of the species *Komagataeibacter hansenii*, which, due to an interesting phenotype and high cellulose yield, became a model organism used for genetic, molecular and biochemical studies (Florea, *et al*., 2016b). The number of reports of bacteria producing BNC continues to grow, as well as the annotation of the putative operons of cellulose synthase in whole bacterial genome sequences, suggesting that more and wider groups of bacteria may be capable of producing cellulose (Solano *et al*., 2002; Chawla *et al*., 2009; Esa *et al*., 2014; Matsutani *et al*., 2015; Keshk and El‐Kott, 2016; Moniri *et al*., 2017; Reiniati *et al*., 2017).

Of particular interest in *Komagataeibacter* strains is the study of molecular aspects of BNC biosynthesis. Genetic engineering is a powerful tool being used to produce valuable strains to increase the efficiency of BNC biosynthesis, as will be further presented below.

## Molecular mechanisms of BNC biosynthesis by *Komagataeibacter* genus

The biochemical reactions of BNC biosynthesis by *K. xylinus* are very well characterized (Brown, 1987; Delmer and Amor, 1995). This is a precise and specifically regulated multi‐step process, involving numerous individual enzymes and complexes of catalytic and regulatory proteins. However, their supramolecular structure has not yet been fully defined. The pathways and mechanisms of the synthesis of uridine diphosphoglucose (UDP‐Glc) are relatively well known, while the molecular mechanisms of glucose polymerization in long and unbranched chains still need to be further investigated.

**Figure 1:**
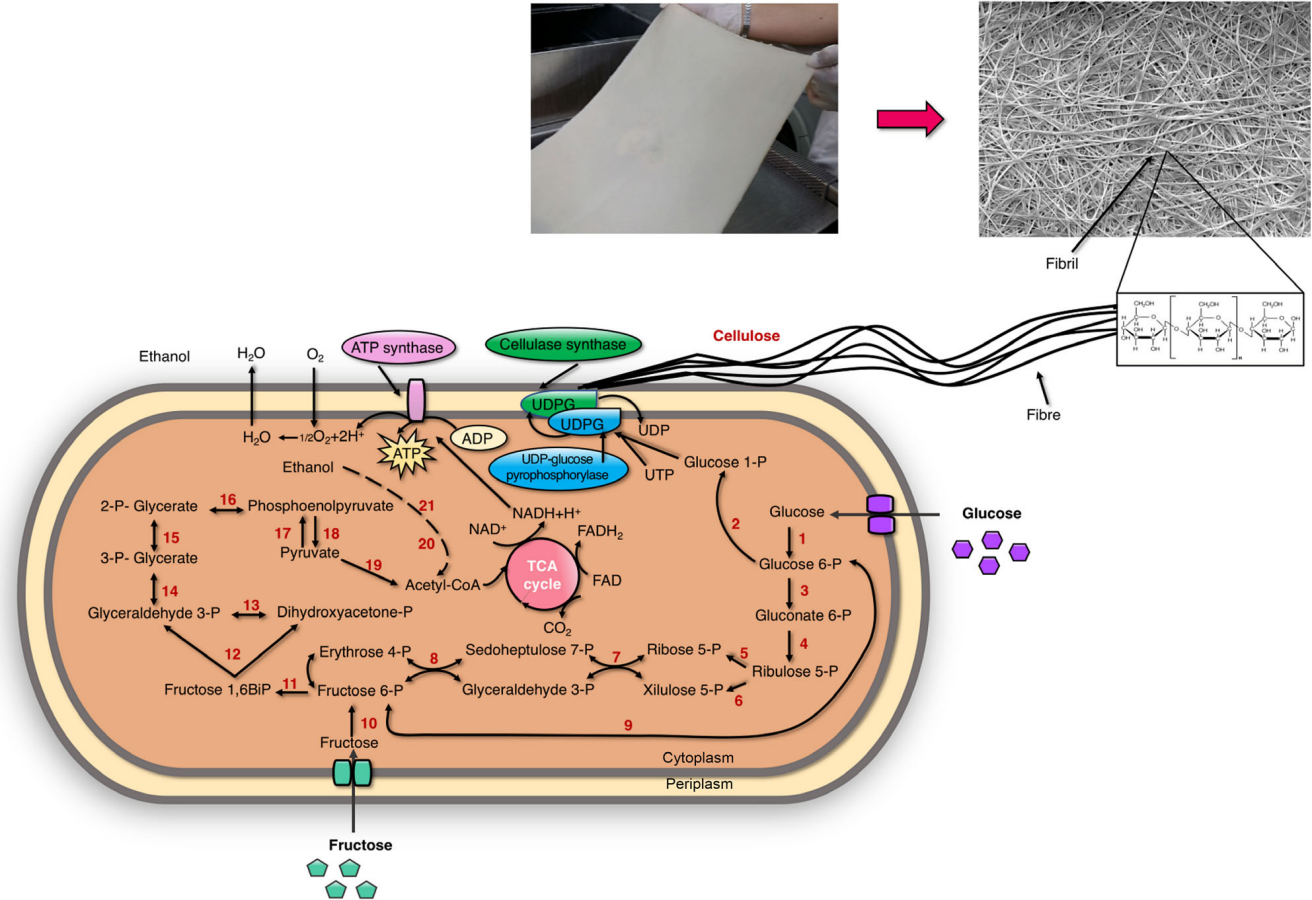
Pathways for the biosynthesis of BNC by *K. xylinus* and assembly of cellulose molecules into nanofibrils: (1) Glucokinase‐ATP, (2) Phosphoglucomutase, (3) Glucose‐6‐phosphate dehydrogenase, (4) 6‐phosphogluconate dehydrogenase, (5) Phosphorribulose isomerase, (6) Phosphorribulose epimeraase, (7) Transaketolase, (8) Transaldolase, (9) Phosphoglucoisomerase, (10) Fructokinase, (11) Fructokinase ATP, (12) Aldolase, (13) Triosephosphate isomerase, (14) Glyceraldehyde 3‐phosphate dehydrogenase, (15) Phosphoglycerate mutase, (16) Enolase, (17) Pyruvate kinase (18) Pyruvate biphosphate kinase, (19) Pyruvate dehydrogenase, (20) Alcohol dehydrogenase and (21) Aldehyde dehydrogenase.

Bacterial nanocellulose is synthesized in two stages, the first being the production of β‐1,4‐glucan chains and the second the crystallization of cellulose (Brown and Saxena, 2000). The conversion of glucose to cellulose requires four enzymatic steps (Fig. 1): phosphorylation of glucose by glucokinase to glucose‐6‐phosphate (G6P); isomerization of glucose‐6‐phosphate to glucose‐1‐phosphate (G1P) by phosphoglucomutase (PGM); conversion of glucose‐1‐phosphate to uridine diphosphate glucose (UDP‐glucose) by UDP‐glucose pyrophosphorylase; and finally, the synthesis of cellulose from UDP‐glucose by cellulose synthase (Bcs), a complex of four subunits, BcsA, BcsB, BcsC and BcsD, which are encoded by three (*bcsAB, bcsC* and *bcsD*) or four (*bcsA, bcsB, bcsC* and *bcsD*) genes (Ross *et al*., 1991; Jedrzejczak‐Krzepkowska *et al*., 2016). UDP‐glucose pyrophosphorylase is a key enzyme in this process, since its activity in cellulose‐producing bacteria is one hundred times higher than in the non‐producing counterparts (Valla *et al*., 1989).

As shown in Fig. 1, the biosynthesis of BNC is closely related to many metabolic pathways, such as the pentose‐phosphate (PP) pathway, Embden–Meyerhof–Parnas (EMP) pathway, the Krebs cycle (TCA) and gluconeogenesis (GNG) (Lee *et al*., 2014; Nagashima *et al*., 2016). Many different compounds, such as hexoses, glycerol, dihydroxyacetone, pyruvate or dicarboxylic acids, can be converted to cellulose. Pyruvate, like glycerol, dihydroxyacetone and intermediates of the pentose monophosphate cycle can be converted into glucose‐6‐phosphate by gluconeogenesis (Krystynowicz *et al*., 2005). On the other hand, disaccharides, such as sucrose or maltose, are first hydrolysed to monosaccharides and then also converted to glucose‐6‐phosphate (Lee *et al*., 2014).

Due to the lack or very low phosphofructokinase (pfk) activity, certain cellulose‐producing bacteria, such as *K. xylinus*, are unable to use EMP pathway for pyruvate synthesis from glucose. Alternatively, pyruvate is obtained from acetate and is used to synthesize glucose through the GNG pathway (Velasco‐Bedrán and López‐Isunza, 2007; Sarkar *et al*., 2010; Zhong *et al*., 2013). The PP cycle involves the oxidation of carbohydrates, and the TCA cycle involves the oxidation of acetate‐derived carbohydrates, fat and proteins, such as oxalosuccinate and α‐ketoglutarate. Nevertheless, *K. xylinus* is not able to metabolize glucose anaerobically since it lacks phosphofructose kinase, which is required for glycolysis (Lee *et al*., 2014).

The model of *Komagataeibacter* metabolism, proposed by Velasco‐Bedrán and López‐Isunza, indicates the connection between catabolic pathways of ethanol, glucose and fructose (Velasco‐Bedrán and López‐Isunza, 2007). The G6P can be metabolized into acetate via phospho‐ketolase pathway, linking glucose and ethanol catabolic pathways. However, ethanol dissimilation feeds the TCA cycle and the production of pyruvate feeds gluconeogenesis through phospho enol pyruvate, connecting ethanol to the glucose pathways. In consequence, all four metabolic products (acetic acid, biomass, acetan and cellulose) may be produced from either of the carbon sources or from a mixture of the two, although the energy balance is different in either case (Velasco‐Bedrán and López‐Isunza, 2007).

Recently Zhong *et al*. (2013, 2014) proposed a similar model of metabolism, but excluded the catabolism of ethanol. In the first study, they clarified as to the higher cellulose yield in *K. xylinus* (CGMCC no. 2955) from glycerol, glucose and fructose by the increased metabolic flux of carbon to BNC. The analysis of the central carbon metabolic flux showed that about 47.96% of glycerol was conveyed into BNC, a value that drops to only 19.05% for glucose and 24.78% for fructose. Whereas, when glucose was used as the carbon source, 40.03% of glucose was turned into the by‐product gluconic acid (Zhong *et al*., 2013). Furthermore, the same authors demonstrated that formation of gluconic acid determines BNC productivity (Zhong *et al*., 2014). These studies indicate that both carbon and energy metabolism influence BNC yields.

The metabolic flux analysis conducted by Li *et al*. (2016) showed that EMP pathway in *K. hansenii* was fully active. Moreover, the low phosphofructokinase activity was detected in *K. oboediens* and this activity was significantly increased along with the gluconate feed concentration (Sarkar *et al*., 2010).

## Characterization of proteins involved in the BNC biosynthesis

**Figure 2:**
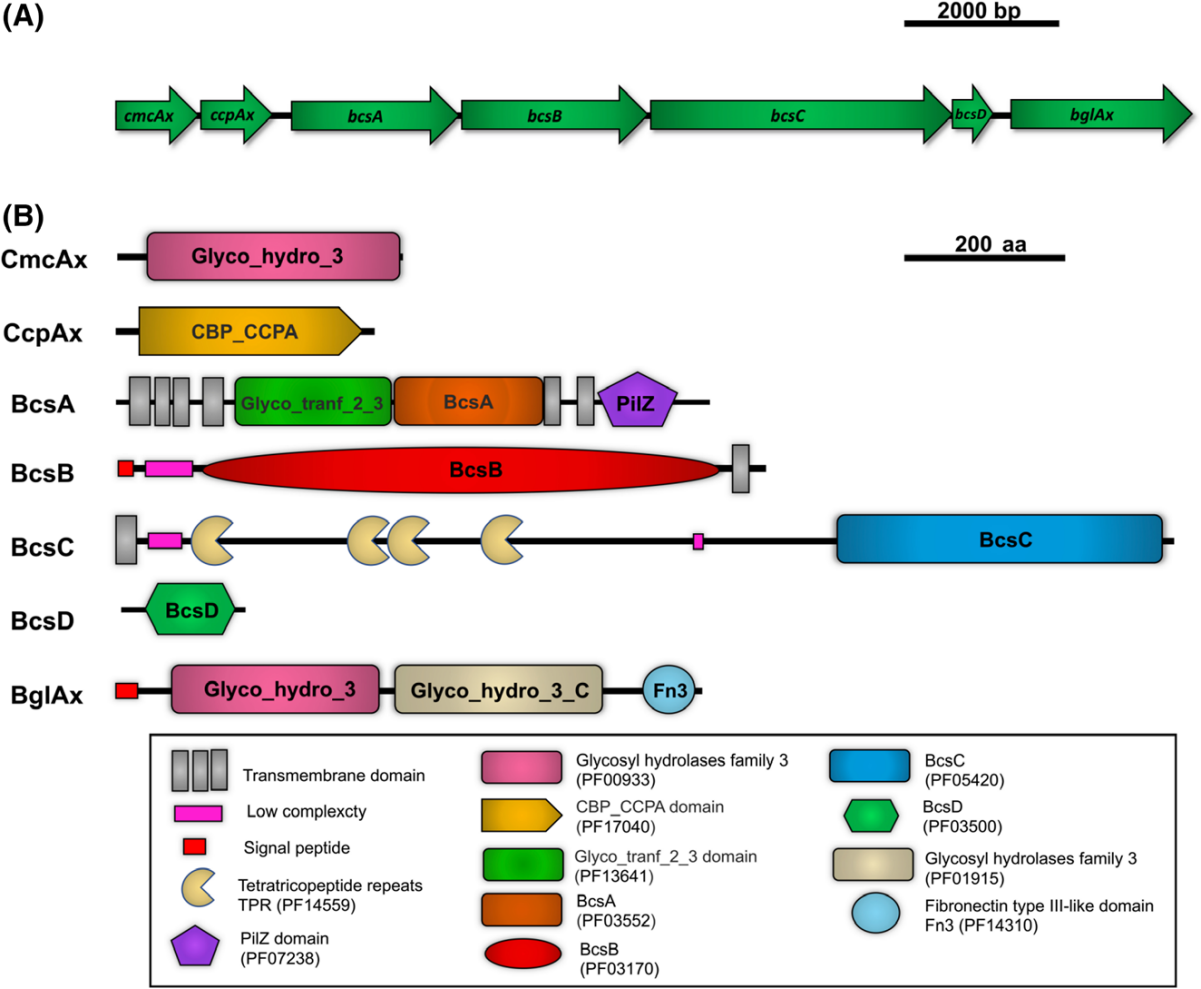
A. Organization of cellulose synthase operon and its flanking regions in Komagataeibacter xylinus E25 Accession no. CP004360 (Ia, locus tags H845_449 → H845_455).B. A cartoon showing the domain organisation of cellulose synthase operon and its flanking regions in Komagataeibacter xylinus E25. Domains were identified by a combined use of Blast (Altschul *et al*., 1997); HMMER/Pfam (Bateman *et al*., 1999); and SMART (Schultz, 2000).

The essential enzyme machinery involved in BNC biosynthesis includes cellulose synthase and endo‐1,4‐glucanase (CMCax) flanking genes, a complementary cellulose factor (CcpAx) and β‐glucosidase (BglAx). Cellulose synthase is an enzymatic complex containing three (*bcsAB, bcsC, bcsD*) or four (*bcsA, bcsB, bcsC, bcsD*) subunits encoded by genes found in the *bcs* (*bacterial cellulose synthase*) operon (Fig. 2). In 1994, the cellulose synthase complex *bcsABCD* was discovered in *K. xylinus* (Saxena *et al*., 1994). The protein subunits included in the cellulose synthase complex and the accessory proteins fulfil various functions identified in Table 1.

**Table 1:** Summary of the function of cellulose synthase subunits and proteins involved in the BNC biosynthesis in bacteria of the genus *Komagataeibacter*

Protein	Function	References
BcsA	Cellulose synthase catalytic subunit	McNamara *et al*. (2015); Römling and Galperin (2015); McManus *et al*. (2016)
	Shows the activity of β‐1,4‐glycosyltransferase	
	Catalysing the polymerization reaction of UDP‐Glucose monomers to β‐1,4‐glucan chains (cellulose precursor)	
	Forms the core of the cellulose synthase complex	
BcsB	Forms the core of the cellulose synthase complex	McNamara *et al*. (2015); Römling and Galperin (2015); McManus *et al*. (2016)
	Takes part in the transport of the newly synthesized β‐1,4‐glucan chain from the cytoplasm through the periplasmic space	
BcsC	Probably creating pores in the outer cell membrane and is involved in the export of the synthesized polysaccharide outside the cell	McNamara *et al*. (2015); Römling and Galperin (2015); McManus *et al*. (2016)
BcsD	It is probably responsible for the formation of crystalline regions of the cellulose chain by facilitating hydrogen formation bonds between the four newly established chains of β‐1,4‐glucan	McNamara *et al*. (2015); Römling and Galperin (2015); McManus *et al*. (2016)
CMCax	It exhibits β‐endo‐1,4‐glucanase activity	Römling and Galperin (2015); Castiblanco and Sundin (2016); McManus *et al*. (2016)
	Takes part in the regulation of packing cellulose fibrils	
	Literature reports indicate that the BcsZ protein is an active participant in the activation of cellulose biosynthesis by c‐di‐GMP	
CcpAx	A protein specific to acetic bacteria	McManus *et al*. (2016)
	It probably interacts with the BcsD subunit	
	The effect of this protein on the activity of cellulose biosynthesis in *K. xylinus* and *K. hansenii* was noted	
BglxA	An enzyme with β‐glucosidase activity	McNamara *et al*. (2015); Römling and Galperin (2015)
BcsX	It probably has cellulose deacetylase activity	McNamara *et al*. (2015)
BcsY	Possible cellulose transacylase	McNamara *et al*. (2015)
	Creating modified polysaccharides, e.g. acetylocellulose	

**Figure 3:**
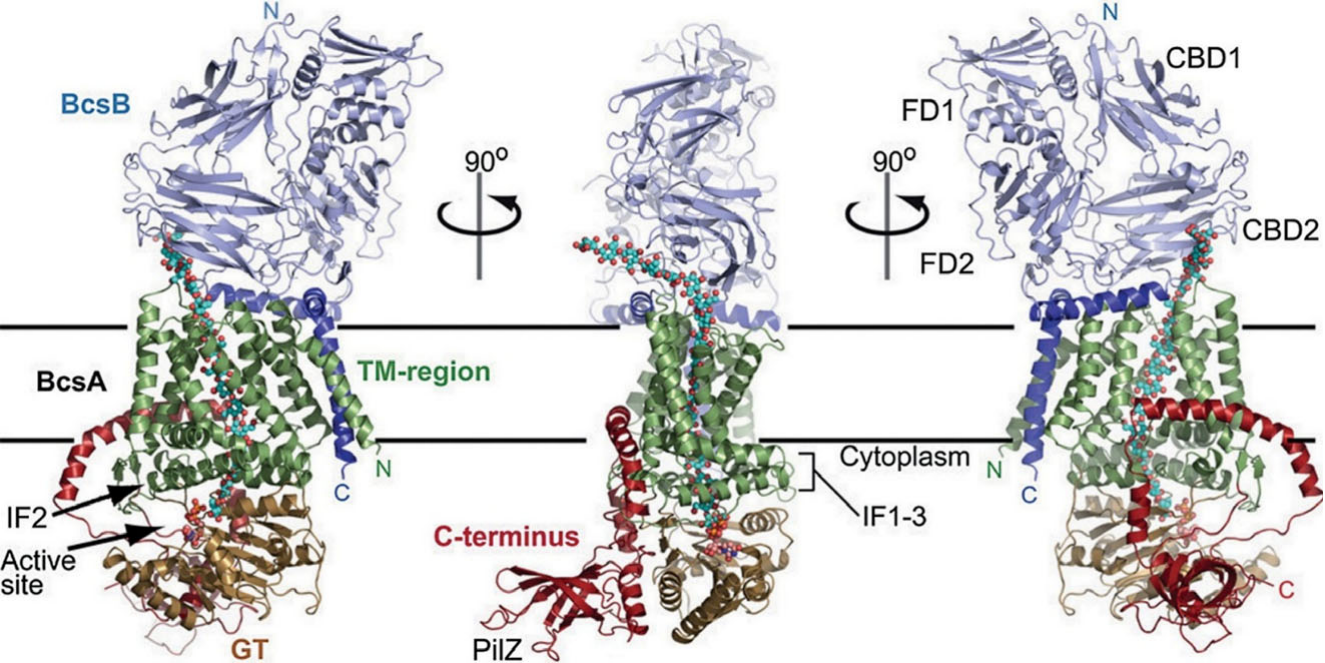
Crystal structure of the BcsA–BcsB complex (Morgan *et al*., 2013).

The crystallographic structure of the BcsA and BcsB subunits of the cellulose synthase complex from *Rhodobacter sphaeroides* was resolved for the first time in 2013 (Morgan *et al*., 2013). The BcsA and BcsB subunits form the core of the cellulose synthase complex, anchored in the inner cell membrane. This core is extremely important for the production of BNC, both *in vivo* and *in vitro* (Römling and Galperin, 2015). The BcsA subunit is associated with the cytoplasmic membrane through 8 TM helices flanked by three amphipathic helices that run parallel to the membrane at the cytosolic water–lipid interface (Kimura *et al*., 2001; Morgan *et al*., 2013; McNamara *et al*., 2015). The cytoplasmic region of the BcsA subunit consists of a catalytic domain with glucosyltransferase (GT) activity using UDP‐Glc as a precursor of the β‐1,4‐glucan chain and the PilZ regulatory domain, that binds a specific allosteric activator to the c‐di‐GMP molecule (Fig. 3). The GT domain of BcsA contains the conserved signature D,D,D,Q (Q/R)XRW which is present in each glycosyltransferases that use a nucleotide‐sugar as a glycosyl donor (Morgan *et al*., 2013). The first two conserved Asp residues (D‐D) coordinate UDP, while the third D residue is presumably important for catalysis (Jedrzejczak‐Krzepkowska *et al*., 2016). The Q(Q/R)XRW sequence belonging to IF2 is a part of the cytoplasmic entry to the glucan channel and together with an equally conserved FFCGS sequence, forms a binding site for the terminal disaccharide of the glucan, the acceptor (Morgan *et al*., 2013).

The BcsA subunit is associated with the cytoplasmic membrane through 8 TM helices flanked by three amphipathic helices that run parallel to the membrane at the cytosolic water–lipid interface (Kimura *et al*., 2001; Morgan *et al*., 2013; McNamara *et al*., 2015).

The BcsB subunit is a periplasmic protein bound to the cytoplasmic membrane via the C‐terminal end of a single transmembrane helix. The periplasmic fragment contains two copies of the repeating carbohydrate‐binding domain (CBD) fused to the domain of flagroxin (FD) (McNamara *et al*., 2015). The CBD domains together with the transmembrane helices 3–8 belonging to the BcsA subunit form the export channel of the resulting polysaccharide from the cytoplasmic space (McNamara *et al*., 2015).

The BcsC subunit, the periplasmic protein, is probably responsible for exporting cellulose out of the cell. It contains an N‐terminal α‐helical motif consisting of several tetratricopeptide (TPR) repeats and a C‐end fragment structurally similar to the β‐barrel characteristic of proteins located in the outer cell membrane (Römling and Galperin, 2015).

The BcsD subunit is a homo‐perceptic periplasmic protein probably involved in the formation of crystalline regions in the newly formed cellulose chain. The functional protein consists of four dimers: AB, CD, EF and GF, forming a cylindrical structure with an internal channel able to host four β‐1,4‐glucan chains. These chains, due to mutual interaction, undergo rearrangement, creating a crystalline region with a strictly defined structure (Hu *et al*., 2010).

The cellulose synthase operon contains two genes in the upper region, *cmcax* and *ccpAx*, and one in the lower region, *bglAx*. The *cmcax* gene encodes endo‐β‐1,4‐glucanase, a globular protein belonging to the cluster I in the family of 8 glycoside hydrolases. The CMCax structure consists of eleven α‐helixes and seven β‐strands (Yasutake *et al*., 2006). CMCax exhibits hydrolytic activity with respect to cellulose and affects the regulation of biosynthesis (Kawano, *et al*., 2002a; Kawano, *et al*., 2002b). It has been shown that in small amounts, exogenous CmcAx enhances BNC production of *K. xylinus*, while endogenous overexpression of *cmcAx* also enhanced the yield of BNC production (Augimeri *et al*., 2015).

The second protein found in the upper region of the *bcs* operon is CcpAx, (*Cellulose complementing protein Acetobacter xylinum*) also called Ccp (*Cellulose complementing factor)* (Deng *et al*., 2013). Research conducted by Standal *et al*. (1994) showed that the *ccpAx* gene plays an important role, because mutants with disruption of the *ccpAx* gene did not produce cellulose *in vivo*. In turn, Sunagawa *et al*. (2013) demonstrated, by means of fluorescence microscopy, that CcpAx is colocalized with BcsD along one side of the cell, and furthermore, using pulldown analysis and the isothermal method of calorimetric titration, that CcpAx and BcsD interact with each other. Although these results indicate that CcpAx may act as a mediator of protein‐protein interactions, its exact role in cellulose biosynthesis is still unknown.

In the lower region of the BNC synthase operon, there is a *bglAx* gene coding for β‐glucosidase, which belongs to the family of three glycoside hydrolases (GH3). BglxA is secreted and has the ability to hydrolyse oligosaccharides larger than three residues to single β‐D‐glucose units (Tahara *et al*., 1998; Tajima *et al*., 2001). It was also shown that BglAx from *K*. *xylinus* BPR2001 has exo‐1,4‐β ‐glucosidase activity and probably also glucosyltransferase activity (Tahara *et al*., 1998). Although BglAx is not essential for the production of BNC, the *bglAx* disruption mutant of *K. hansenii* ATCC 23769 produced less cellulose than the wild‐type strain. The expression of the *bglAx* gene has been shown to be regulated transcriptionally by the cyclic‐AMP/fumarate nitrate reductase (CRP/FNRKh) receptor. In contrast, the transposon insertion into crp/fnrKh completely abolished the production of BNC and BglAx, proving that CRP/FNRKh controls the biosynthesis at the transcription level (Deng *et al*., 2013).

## Regulation of the biosynthesis of BNC

The BNC biosynthesis is regulated at both the transcriptional and post‐translational levels (Zogaj *et al*., 2001). A well‐known regulatory mechanism for this process is the allosteric activation of BcsA by cyclic diguanylate (c‐di‐GMP), a universal bacterial second messenger discovered in *K. xylinus* (Ross *et al*., 1990; Römling, 2012). In *Komagataeibacter,* the cellulose biosynthesis is modulated by the opposing action of two enzymes, diguanylate cyclase (DGC) and c‐di‐GMP diesterase (PDEA), controlling cellular levels of c‐di‐GMP (Ross *et al*., 1987; Tal *et al*., 1998). Cyclic‐di‐GMP is produced from two GTP molecules by DGC, whose activity is associated with the conserved (GGDEF) domain (Ausmees *et al*., 2001; Chang *et al*., 2001). Specific phosphodiesterases (PDEs) that contain EAL or HD‐GYP domains hydrolyse c‐di‐GMP into 5′‐phosphoguanylyl‐(3′‐5′)‐guanosine (pGpG) or GMP, respectively. The oligoribonuclease Orn, which is a ribonuclease that hydrolyses RNAs that are 2–5 nucleotides in length, is the primary enzyme that is capable of degrading pGpG to GMP (Cohen *et al*., 2015; Orr *et al*., 2015). The HD‐GYP domain is a subset of a larger HD family which members have hydrolytic activity for various substrates (Jenal *et al*., 2017).

**Figure 4:**
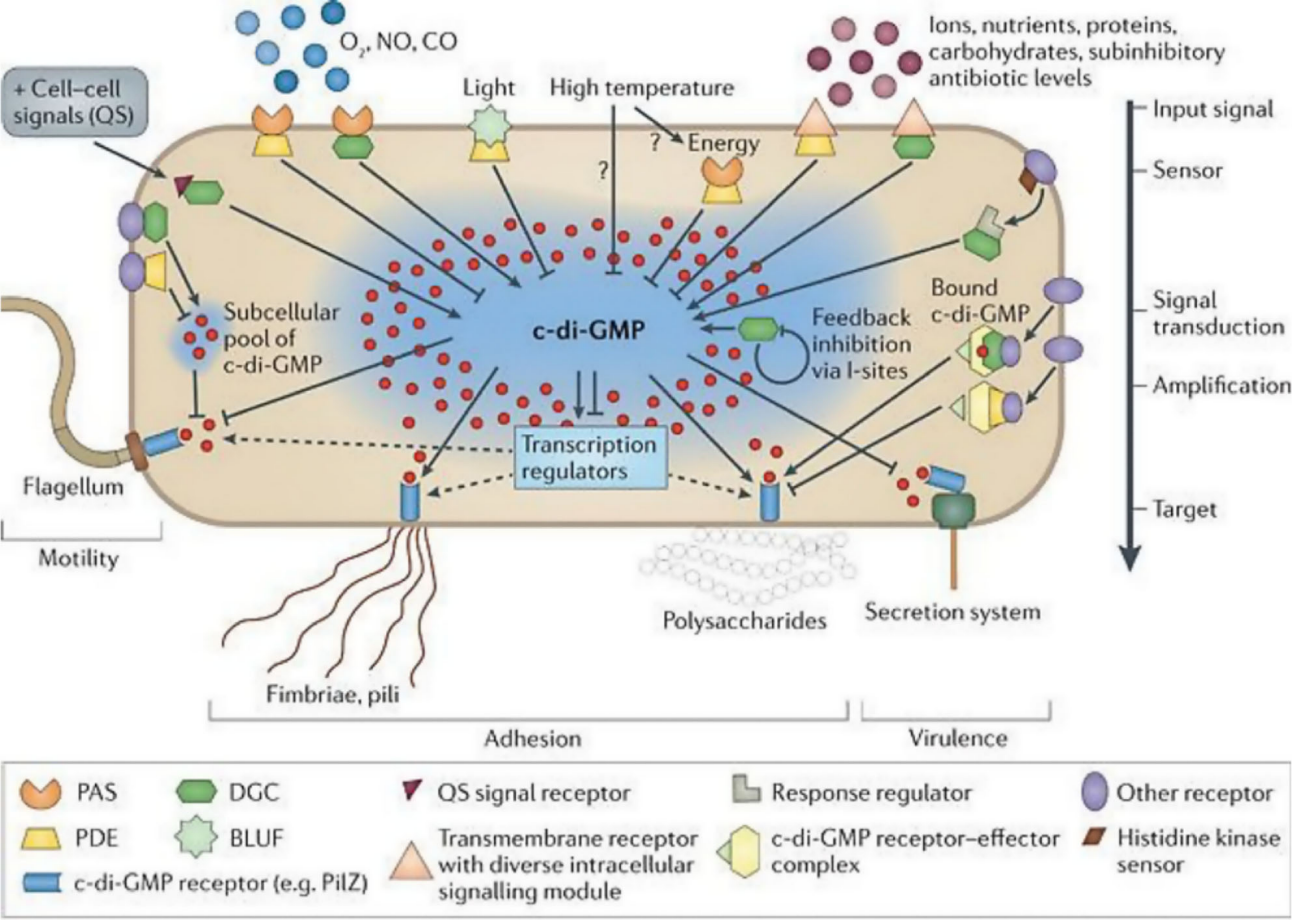
The influence of environmental factors on the biological functions of the bacterial cell (McDougald *et al*., 2012).

Cyclic‐di‐GMP exerts its activity by signalling pathways regulating *e.g*. biofilm formation, flagella biosynthesis, motility, virulence, the cell cycle, bacterial cell differentiation and other fundamental physiological processes in several bacterial species (Castiblanco and Sundin, 2016; Valentini and Filloux, 2016). It plays a key role in the pathways responsible for the conversion from a planktonic to a sessile lifestyle. High intracellular c‐di‐GMP content enhances biofilm formation via the reduction of motility and production of biofilm matrix, whereas low intracellular c‐di‐GMP content leads to increased motility, biofilm dispersal and the return to planktonic phase (Dow *et al*., 2007; Schirmer, 2016; Lin Chua *et al*., 2017). As a bacterial second messenger, it mediates signals coming from the environment onto regulation of many cellular processes (Fig. 4) (Fu *et al*., 2018; Hall and Lee, 2018). Intracellular c‐di‐GMP levels are balanced by the antagonistic activities of DGCs and PDEs, which are frequently found in multiple copies, and which are related within amino‐terminal sensor domains, such as PER–ARNT–SIM (PAS) for sensing gaseous ligands, and blue light using flavin (BLUF) for sensing light (Fig. 4) (Tarutina *et al*., 2006; Tschowri *et al*., 2009).

**Figure 5:**
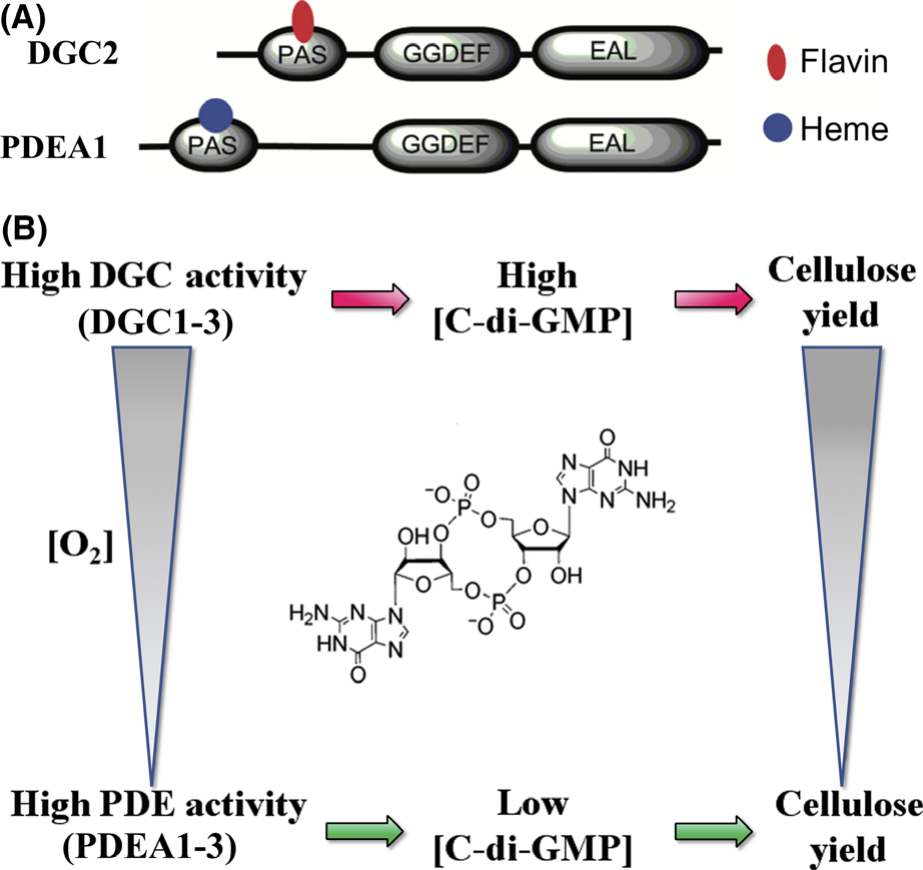
A. Comparison of the domain organization of the PAS domain‐containing proteins in DGC2 and PDEA1 from *K. xylinus*.B. The connection among oxygen level, cellular c‐di‐GMP concentration and cellulose yield in *K. xylinus* based on Qi *et al*. (2009).

The PAS domain is one of the most common and well‐known sensor domains, which receives environmental signals such as: light, redox potential and oxygen concentration (Vogt and Schippers, 2015). It has been identified in numerous signalling proteins located in the cytoplasm. Depending on the enzyme PAS is associated with, it may bind to different cofactors, e.g. oxygen or the oxidized form of FAD (Dow *et al*., 2007). Oxygen level sensing was first reported in *K. xylinus* for the PDEA1 haem‐binding PAS domain (Chang *et al*., 2001), where it regulates c‐di‐GMP hydrolysis by reversibly binding O_2_ and through conformational changes in the EAL catalytic domain. These findings provide additional evidence of the significance of oxygen availability for appropriate c‐di‐GMP level regulation and, in consequence, cellulose biosynthesis activation in *K. xylinus*. Further research revealed that *K. xylinus* diguanylate cyclase 2 (DGC2) contains the PAS domain that binds a flavin adenine dinucleotide (FAD) cofactor noncovalently (Qi *et al*., 2009). Binding of the oxidized form of flavin nucleotide regulates the activity of the GGDEF domain and therefore the c‐di‐GMP synthesis. Together with the regulation of PDEA1 by O_2_, these findings also underline the regulation of c‐di‐GMP concentration and cellulose biosynthesis through both heme‐ and flavin‐containing PAS domains and O_2_ sensing in *K. xylinus* (Fig. 5) (Chang *et al*., 2001; Qi *et al*., 2009). These studies provide preliminary molecular elucidation of the role of O_2_ concentration in the regulation of cellulose synthase activity by c‐di‐GMP in *K. xylinus*, although direct evidence for this correlation is still missing.

Due to the pioneer work of Professor Moshe Benziman, the isolation of the *cdg1*,* cdg2* and *cdg3* operons derived from *K. xylinus*, which encode homologous isoforms of DGC and PDEA, was performed (Ross *et al*., 1986, 1987). It has been shown that each operon is organized into the *pdea* gene located above the *dgc* gene. Genetic analyses among *dgc* operons indicate that they contribute in different ways to the PDEA and DGC enzymatic activity (*cdg1* is responsible for 80% of each activity, while *cdg2* and *cdg3* account respectively for 15% and 5%). The discovery of the three *cdg* operons reveals the unusual genetic organization of bacteria. In the genomes of *K. hansenii* ATCC 23769 and *K. hansenii*, ATCC 53582 two Cdg operons (*cdg1* and *cdg2*) containing a diguanylate cyclase gene (*dgc1, 2*) and phosphodiesterase A (*pdeA1, 2*) as well as four standalone c‐di‐GMP phosphodiesterases (*pdeA3–6*) were found. Interestingly in *K. xylinus,* there are three operons encoding enzymes regulating the level of c‐di‐GMP (Tal *et al*., 1998; Deng *et al*., 2013; Florea *et al*., 2016b). The arrangement of genes encoding enzymes with opposite actions within the same genetic unit is rare. In addition, the proteins encoded by the *dgc* and *pdeA* genes show a high degree of identity within each isoenzyme package and significant structural conservation. The N‐terminus of all six isoenzymes contains domains similar to those found in various oxygen‐sensing proteins. Furthermore, the DGC and PDEA sequences share a long motif, consisting of two adjacent areas defined by GGDEF and EAL. Coordinated expression of *pdeA* and *dgc* provides the necessary balance of PDEA and DGC to achieve optimal c‐di‐GMP concentration, which is essential for the rate of cellulose biosynthesis in accordance with environmental conditions (Tal *et al*., 1998).

**Figure 6:**
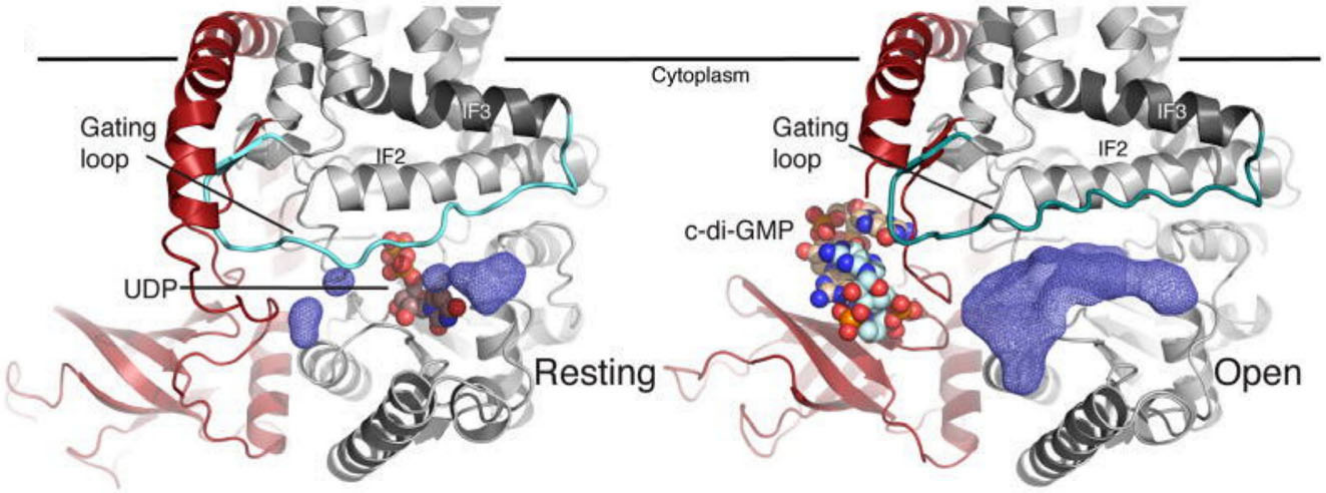
Gating loop positions in the absence and presence of c‐di‐GMP (Morgan *et al*., 2014).

The mechanism of activation of BNC production is relatively simple and consists in post‐translational modification of the BcsA subunit. BcsA without the associated allosteric agent is catalytically inactive and unable to conduct the polymerization reaction (Morgan *et al*., 2014). In the free state, the gating loop blocks the access of the substrate (UDP‐Glc) to the glucosyltransferase domain (GT) (Fig. 6). The binding of c‐di‐GMP through the PilZ regulatory domain results in a number of conformational changes leading to the exposure of the active site.

The C‐terminal PilZ domain contains two motifs: an ‘RXXXR’ with conserved arginine residues surrounding one guanine base of c‐di‐GMP, and a ‘DxSxxG’ motif that surrounds the other guanine of the molecule (Whitney *et al*., 2015; Chou and Galperin, 2016). The binding of c‐di‐GMP dimer is necessary for the allosteric activation of the BcsA subunit (Castiblanco and Sundin, 2016). The first molecule (c‐di‐GMP‐A) interacts with the ‘DxSxxG’ motif, while the second one (c‐di‐GMP‐B) is stabilized by π–π stacking interactions with c‐di‐GMP‐A (Morgan *et al*., 2014). Binding of two molecules of the cyclic di‐GMP results in a change in the position of the gating loop and the exposure of the UDP‐Glc binding site in the GT domain, consequently activating the cellulose synthase (Morgan *et al*., 2014).

It was shown that the GGDEF domain is responsible for the synthesis of c‐di‐GMP thanks to its diguanylate cyclase activity, whereas the EAL domain is responsible for its degradation through its phosphodiesterase activity (Paul *et al*., 2004). In some cases, only one of those domains is active in GGDEF‐EAL proteins, as it seems to be the case for the three *K. xylinus* proteins with conserved GGDEF domains, but distinct EAL domains from the active phosphodiesterase A of the same strain (Römling *et al*., 2005; Schmidt *et al*., 2005). However, some *in vivo* observations suggest that some GGDEF‐EAL proteins may have both DGC and PDE activity. For example, the disruption of diguanylate cyclase 1 (dgc‐1), which is responsible for 80% of the c‐di‐GMP production in *K. xylinus*, caused a reduction in BNC production under certain growth conditions, but a surprisingly increased production under different conditions (Tal *et al*., 1998; Bae *et al*., 2004). Therefore, additional work is needed to determine the full enzymatic capacity of proteins containing both the GGDEF and EAL domains.

The availability of high‐resolution crystal structures of the domains GGDEF, EAL and HD‐GYP combined with site‐directed mutagenesis studies allows for the formulation of general principles of differentiation domains. In the GGDEF domain, the active site (*A site*) contains catalytic Asp/Glu residues surrounded on each side by two highly conserved residues that together form ^79^GG (D/E) EF^83^ (amino acid residue numbering is derived from the GGDDEF domain of the NCBI *Conserved Domain Database*). The first two glycines are involved in GTP binding, while the fourth Glu is involved in the coordination of metal ions. In addition, the active site includes an Asp38 residue that binds Mg^2+^, as well as Asn46 and Asp55, which binds guanine. Indeed, the RYGGEEF active site motif found in such proteins as PleD, WspR and HmsT requires the presence of conserved residues surrounding the catalytic Glu81. The mutated HmSt form from *Y*. *pestis* with the changed motif in the RYAGEEF active centre is not active. In contrast, the motif ‘RxGGDEF’ with Asp81 catalytic residue can contain a variety of hydrophobic residues at position ‘x.’ In addition, DGC activity also remains when the first Glu is replaced with Ala or Ser. In the position of five amino acid residues above the GG (D/E) EF domain, allosteric inhibitory place of the so‐called I site consists of four residues RxxD, where x stands for each residue. It has been demonstrated that the allosteric c‐di‐GMP binding site (I site) is responsible for the non‐competitive inhibition of the DGC product. It has been observed that place I is conserved in the majority of known and potential DGC proteins (Christen *et al*., 2006; Romling *et al*., 2013).

It was also reported that the quorum‐sensing (QS) system positively regulate phosphodiesterase, which decompose c‐di‐GMP (Liu *et al*., 2018). Quorum‐sensing *is a* cell‐to‐cell signalling system that controls bacterial social behaviours, such as biofilm formation, virulence and motility (An *et al*., 2014). Quorum sensing controls density‐dependent gene expression by the secretion and detection of chemical signals called QS autoinducers to sense the local population density (Ng and Bassler, 2009). Many gram‐negative bacteria were reported to use a different class of autoinducers: the acyl‐homoserine lactones (AHLs) (Srivastava and Waters, 2012). In *Komagataeibacter intermedius*,* pde* expression was shown to be positively regulated by the QS N‐AHL‐dependent system termed GinI/GinR (Iida *et al*., 2008, 2009). Recently, AHLs were identified in *K. xylinus* CGMCC 2955, where they are synthesised by the LuxR–LuxI system (Liu *et al*., 2018). When the population density reaches the ‘quorum,’ AHLs exceed the threshold concentration and bind LuxR, a complex that activates the transcription of a specific operon. Since *luxI* and *luxR* are homologs of *ginI* and *ginR*, and c‐di‐GMP is an activator of the BcsA–BcsB subunit, Liu *et al*. (2018) hypothesized that BNC biosynthesis may be regulated by QS by controlling c‐di‐GMP levels.

Recently, a novel cellulose biosynthesis regulator, the quorum‐quenching protein GqqA, has been described. The addition of recombinant GqqA protein to growing cultures of the *Komagataeibacter europaeus* CECT8546 exerted a strong impact on cellulose production (Valera *et al*., 2016). The density of the culture increased overtime in the presence of the GqqA protein, whereas the control cultures did not display any altered behaviour. (Valera *et al*. (2016) suggested that this effect is attributable to alterations in the produced AHL molecules.

Another group of BNC biosynthesis regulator is the CRP/FNR family transcription factors. CRP/FNR_Kh_ regulates BNC biosynthesis in *K. hansenii* ATCC 23769 through positive regulation of *bglAx* expression (Deng *et al*., 2013). Interestingly, a Crp/Fnr protein in *Burkholderia cenocepacia*, named Bcam1349, binds c‐di‐GMP and regulates biofilm formation by enhancing the production of BNC and curli fimbriae (Fazli *et al*., 2011). Binding of c‐di‐GMP in turn enhanced the ability of Bcam1349 to bind the promoter region and increase the expression of cellulose synthase operon genes, along with the Bcam1330‐Bcam1341 gene cluster involved in exopolysaccharide biosynthesis (Fazli *et al*., 2011). In recent years, Augimeri and Strap (2015) identified a novel phytohormone‐regulated CRP/FNR_Kx_ transcription factor that directly regulates BNC biosynthesis in *K. hansenii* ATCC 53582 at a transcriptional level, similarly to CRP/FNR_Kh_ in *K. hansenii*. Furthermore, they reported the ethylene up‐regulated *crp/fnr*_*Kx*_ expression and enhanced BNC production in *K. hansenii*, directly by up‐regulating the expression of *bcsA* and *bcsB*, and indirectly though the up‐regulation of *cmcAx*,* ccpAx* and *bglAx* (Augimeri and Strap, 2015).

## Genetic instability

**Figure 7:**
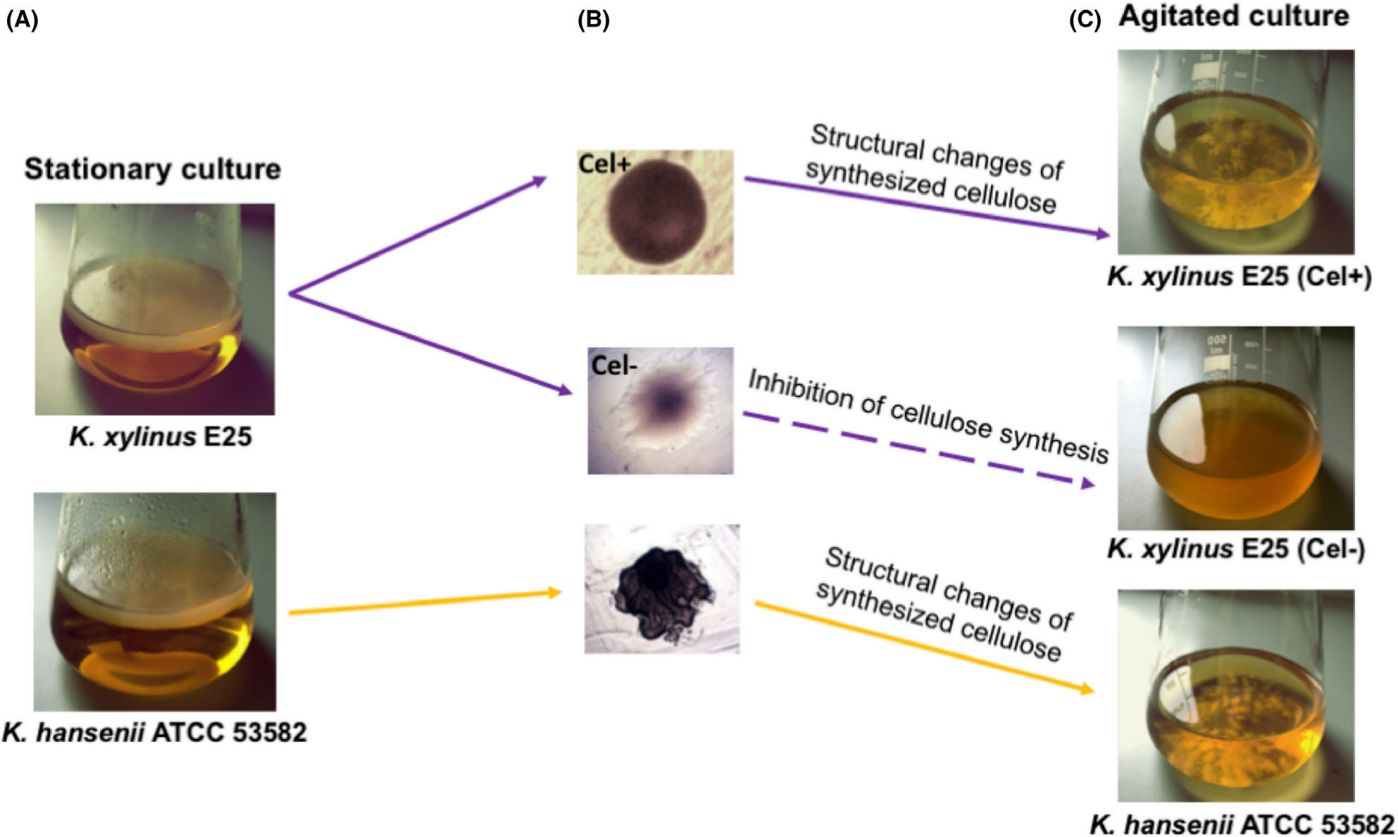
Stable strain of *K. hansenii* ATCC 53582 and unstable strain *K. xylinus* E25 generating Cel− and Cel+ forms.A. Stationary culture – thin homogenous BNC membrane formed on the SH medium surface.B. Microscopic pictures of colonies formed by the *K. xylinus* E25 Cel− and Cel+ forms and *K. hansenii* ATCC53582.C. Agitated culture – cellulose biosynthesis in the form of small beads (*K. hanseii* ATCC 53582 and *K. xylinus* E25 Cel+) or lack of BNC biosynthesis (*K. xylinus* E25 Cel−).

An important obstacle in the implementation of the BNC biosynthesis process to the industry is the huge diversity between the *Komagataeibacter* strains in terms of nutritional requirements and the production efficiency. In addition, for some strains, the formation of spontaneous mutants Cel− (that do not produce cellulose) is observed, especially in submerged cultures (Fig. 7). The occurrence of Cel‐mutants in shaken cultures was first observed by Hestrin and Schramm in 1954. They isolated and described three different types of *K*. *xylinus* cells, according to the morphology and performance of BNC biosynthesis (Schramm and Hestrin, 1954). Group I corresponds to wild‐type cells producing BNC. Group II corresponds to cells that do not produce cellulose (Cel−), but are capable of reverting over culture passages, while Group III includes irreversible morphotypes of Cel− (Schramm and Hestrin, 1954).

It was observed that the Cel+ and Cel− morphotypes are characterized by a different morphology. Cel+ colonies are jelly‐like and convex, with smooth edges, while Cel− colonies are large, flat and with wavy edges (Wong *et al*., 1990) (Fig. 7).

The transformation frequency from Cel+ to Cel− depends on the culture conditions. Cel+ cells dominate in stationary cultures, producing a BNC membrane at the liquid–air interface, where cells find a more oxygen rich environment. It has been shown that homogeneous aeration of liquid culture under agitated conditions favours the spontaneous appearance of Cel−, which become dominant over time. Despite the undertaken research on the emergence of Cel−, this phenomenon has not yet been explained at the molecular level. The search for optimal culture conditions limiting Cel− formation allowed the identification of strategies such as replacing glucose with fructose or enriching the glucose medium with ethanol (2%, v/v), as well as properly selecting the agitation speed (Krystynowicz *et al*., 2002; Jae *et al*., 2005).

It was thought that *K. xylinus* plasmids could have a role in the BNC biosynthesis since these strains usually bear several plasmids of various sizes (from 16 to 300 kb). However, although Cel+ and Cel− cells usually exhibit different plasmid profiles, some BNC‐synthesizing strains devoid of plasmids have been found (Valla and Coucheron, 1983).

It was also reported that insertion elements IS1031 could be involved in an unstable cellulose production by *K. xylinus* (Coucheron, 1991). When IS elements appear in the middle of genes, they interrupt the coding sequence and inactivate the expression of that gene (Siguier *et al*., 2014). A correlation has been found between the presence of the IS1031 insertion element and the Cel− morphotype (Coucheron, 1991). Analysis of the protein profile (2D electrophoresis) of the strain *K*. *xylinus* E25 shows that the Cel− morphotype does not have two key enzymes in the cellulose biosynthetic pathway: phosphoglucomutase and glucose‐1‐phosphate urydilotransferase (Krystynowicz *et al*., 2005). On the other hand, other studies have shown that *K*. *hansenii*, ATCC 23769 Cel− spontaneous mutant, has a local transposition element inserted in *ccpAx* (Deng *et al*., 2013).

In *Komagataeibacter hansenii* ATCC 23769, the IS1031 A insertion sequence and other isoforms, namely IS1031 B, IS1031 C and IS1031 D, were located. Genetic analysis of the genomes of nine *K. xylinus* strains showed that all of them contain insertion sequences belonging to the IS1031 group, present in variable number of copies (Coucheron, 1993). The element with slightly lower homology IS 1032 is responsible for the inactivation of genes involved in the synthesis of acetan (Iversen *et al*., 1994). Nevertheless, the reasons for the appearance of Cel− spontaneous mutants are still unclear.

## Genetic modification of *Komagataeibacter* genus

Despite the long‐term and intensive research involving modifications of culturing conditions, it is still not possible to fully control the biosynthesis and the properties of cellulose produced by *Komagataeibacter* species. To achieve this goal, extensive genetic testing is necessary, which would uncover the molecular relationships between proteins involved directly and indirectly in BNC production.

So far, genetic engineering of *Komagataeibacter* strains concerned attempts to increase the efficiency of BNC biosynthesis or to achieve structural changes in the BNC network, hence giving this nanomaterial new properties. These genetic modifications included the expression of a foreign gene or the gene disruption (Edwards *et al*., 1999; Chien *et al*., 2006; Yadav *et al*., 2010).

The most commonly used method of transforming *Komagataeibacter* is by electroporation, which consists in the formation of unstable pores in the cell membrane under the influence of an electric field. The presence of pores allows the penetration of macromolecules present in the intercellular space into the cells (Wong *et al*., 1990; Edwards *et al*., 1999; Chien *et al*., 2006; Yadav *et al*., 2010). Another method of transformation described for *K. xylinus* is the conjugation with *E. coli* cells (Battad‐Bernardo *et al*., 2004). For the expression or overexpression of the gene, replicating plasmids in *K*. *xylinus* cells such as pLBT, pBBR122, pTI99, pBAV1C and the pSA19 shuttle plasmid are used as vectors (Nakai *et al*., 1999; Chien *et al*., 2006; Fang *et al*., 2015; Mangayil *et al*., 2017). Nevertheless, the case of using the transposon mutagenesis using the mini‐Tn10 transposon has also been described (Battad‐Bernardo *et al*., 2004). When the transformation goal is to disrupt one of the genes found in the bacterial chromosome, the corresponding sequence is introduced using a non‐replicating plasmid in *K*. *xylinus* cells, e.g. pACYC184, pKE23, BPR2001, pET‐14b (Saxena *et al*., 1994; Ishida *et al*., 2002; Shigematsu *et al*., 2005; Deng *et al*., 2013, 2015; Kuo *et al*., 2015). The introduced sequence includes the gene of interest interrupted by, e.g., an antibiotic resistance gene. Since the introduced plasmid does not replicate in *K. xylinus* cells, antibiotic resistant cells must arise from homologous recombination and the conversion of the chromosomal gene sequence into the one carried by the plasmid. Gene expression is thus suppressed in these cells due to its interruption (Edwards *et al*., 1999; Yadav *et al*., 2010).

### Inhibition of gene expression

The abolishment of gene expression through disruption was used for the first time in 1990 during the research on the cellulose synthase operon and the function of its genes. For this purpose, the plasmid pACYC184 was used, into which the *bcsD* gene sequence was cloned, and then interrupted using the ampicillin resistance gene sequence. Obtained mutants showed reduced cellulose synthesis efficiency by about 40%, hence being concluded that the *bcsD* gene is required for maximum efficiency of biopolymer synthesis *in vivo* (Wong *et al*., 1990).

However, further research carried out by Saxena *et al*. (1994) showed that the disrupted *bcsD* mutant produced reduced amounts of two cellulose alomorphs (cellulose I and II), suggesting that the *bcsD* gene is involved in the cellulose crystallization. It has been proposed that the BcsD protein is included in the pore complex or involved in the organization of pores of the linear terminal complex (TC). Additional TEM observations of the mutant cells revealed abnormalities in the orientation of the linear TCs on the BcsD mutant cells, while the components within each liner TC appeared to be aligned normally and functioned similar to that of the wild type (Mehta *et al*., 2015). Furthermore, results obtained by Mehta *et al*. (2015) strongly suggest that BcsD aids in the proper orientation of the linear terminal complexes along the longitudinal axis of the cell, indicating a role of this protein in the final level of the hierarchical assembly of cellulose resulting in highly efficient cellulose biosynthesis.

Bae *et al*. (2004) genetically modified the *K*. *xylinus* BPR2001 strain to compare the performance and structural characteristics of BNC produced by mutants with *dgc1* gene disruption with the one obtained from the wild‐type strain. Since the *dgc1* gene plays an important role in the activation of BNC biosynthesis through c‐di‐GMP, its disruption should reduce the production. However, it was found that the disruption of the *dgc1* gene had no apparent effect on BNC production but had a strong impact on its structure. The BNC membranes produced by *dgc1* mutant were characterized by smaller and more dispersed fibres that do not form a characteristic compact, hydrated membrane (Bae *et al*., 2004).

Another modification concerns the disruption of the *aceP* gene encoding the glycosyltransferase involved in the synthesis of the extracellular branched heteropolysaccharide acetan. This is a water‐soluble polysaccharide produced by *Komagataeibacter xylinus* from UDP‐Glucose. The *K. xylinus CHE5* disrupting mutant synthesized acetan with altered structure and also showed unchanged yield of its production (Edwards *et al*., 1999). In the following years, Ishida *et al*. (2002) created a mutant that did not produce acetan (EP1) by disrupting the *aceA* gene in *K. xylinus* BPR2001*,* a gene encodes the β‐glucosyltransferase responsible for the first step of the acetan biosynthetic pathway. Although inhibition of acetan production was expected to increase the concentration of UDP‐Glucose, and thus the yield of BNC, the EP1 mutant produced less BNC under shaking conditions, than the wild‐type strain. After 2 days, the culture medium with the EP1 mutant became a heterogeneous suspension containing large flocs formed by cell aggregates and BNC. Furthermore, the addition of water‐soluble polysaccharides, such as acetan or agar, improved the dispersion of the culture medium and the number of free cells. The authors suggested that the lack of acetan reduces the viscosity of the culture medium and increases the agglomeration of cells and BNC, which in turn led to a reduction in BNC production (Ishida *et al*., 2002).

A further example is the disruption of the *ghd* gene sequence coding for glucose dehydrogenase (GDH) with the chloramphenicol resistance gene in *K. xylinus* BPR2001 cells. The GDH activity is responsible for the extracellular conversion of glucose to gluconic acid, which thus is no longer available as a substrate for the production of BNC in the mutant strain. The GDH coding gene was inserted into the pT7‐Blue T‐Vector plasmid vector and then discontinued with the cat‐1 chloramphenicol acetyltransferase gene sequence. The obtained mutant did not show GDH activity; moreover, it was characterized by a higher efficiency of cellulose biosynthesis (Shigematsu *et al*., 2005). In addition, Kuo *et al*. (2015) showed that the mutant (GDH‐KO) *K*. *xylinus* BCRC 12334 with the disruption of the *gdh* gene produced more BNC as compared to the wild‐type strain (40% under stationary conditions and 230% under shaken conditions). The authors suggested that such a significant increase in the production of BNC in shaking culture is the result of a better mass transfer of O_2_ and nutrients. Kuo *et al*. (2015) further demonstrated that the GDH‐KO mutant can use glucose to produce BNC without producing gluconic acid as a by‐product.

### Gene overexpression

Recently, Mangayil *et al*. (2017) successfully overexpressed the *bcsA* genes, *bcsAB* and a complete cellulose synthase operon (*bcsABCD*) in *K. xylinus* DSM 2325. Although there were no significant differences between the growth of mutants and the wild‐type strain, mutants showed faster production, generating twofold to fourfold more BNC. The highest efficiency was observed in the case of the *bcsABCD* overexpression mutant.

Kawano *et al*. (2002a) demonstrated that overexpression of the *cmcax* gene in *K. hansenii* ATCC 23769 increases the yield of BNC; furthermore, the addition of CMCax protein to the culture medium also promotes the production of cellulose. These authors revealed that the production of BNC may be controlled by regulating the expression of the *cmcax* gene. Another evidence of the impact of CMCax on BNC biosynthesis was studied by Nakai *et al*. (2013) The *K. xylinus* BPR 2001 mutant with disruption of the *cmcax* gene produced less BNC than the wild‐type strain. Also, these authors observed that the mutant produced mainly cellulose II, thus indicating that CMCax affects the crystallinity of cellulose. Electron microscopy results of Kawano *et al*. (2002a) showed that CMCax can affect the cellulose fibre structure. Furthermore, cellulose fibres secreted from the *K*. *hansenii* strain of ATCC 23769 overexpressing the *cmcax* gene produced a relaxed structure compared to wild‐type strains.

### Expression of foreign genes in cells of bacteria of the genus *Komagataeibacter*

A successful example concerns the introduction of a mutant sucrose synthase gene derived from Mung bean into the cells of *K. xylinus* strain BPR2001. Sucrose synthase is an enzyme found in plants that catalyses the reversible reaction of sucrose synthesis with UDP‐glucose and fructose. The gene – under the control of the lac promoter – was introduced into bacterial cells using the pSA19 shuttle plasmid. A gene mutation, involving the replacement of serine at position 11 with glutamic acid, was expected to result in enzyme activation for the synthesis of UDP‐glucose from sucrose and UDP. Indeed, the obtained transformants showed an increase in the content of UDP‐glucose and a twofold and even threefold increase in the synthesis of BNC yield (Nakai *et al*., 1999).

Another genetic modification of *K*. *xylinus* described in the literature concerned the preparation of a strain able to grow on a medium containing lactose as a carbon source, therefore allowing the conversion of cheese whey into BNC. Thus, the promoter‐free lacZ gene coding for β‐galactosidase, one of the genes of the lactose operon, was introduced into the chromosomal DNA of the wild‐type *K. xylinus* ITDI 2.1 strain. For this purpose, the plasmid pLBT containing the transposon mini‐Tn10 and the transposase gene outside the tansposon sequence was used. The mini‐Tn10 sequence includes the lacZ gene and the gene coding for kanamycin resistance flanked by repeated sequences recognized by transposase (Battad‐Bernardo *et al*., 2004; Vizváryová and Valková, 2004). The mutagenization process was performed by conjugation of the *E*. *coli* S17.1 strain, acting as a vector donor, and the wild strain *K*. *xylinus*. Among the obtained mutants, the one showing the highest efficiency of cellulose synthesis was selected. The obtained *K. xylinus* ITz3 strain showed the stability of mini‐Tn10 transposon insertion into the chromosomal DNA and the ability to synthesize the biopolymer on lactose containing medium, as well as on the whey substrate itself. The β‐galactosidase gene was constitutively expressed, resulting in high enzyme activity (Battad‐Bernardo *et al*., 2004).

Another interesting modification regards the introduction of the *vgb* gene present in bacteria of the genus *Vitreoscilla*, which encodes bacterial haemoglobin (Vhb), into *K. xylinus* BCRC 12334 cells. In *Vitreoscilla* cells, VHb synthesis increases with the decrease in extracellular oxygen concentration. This protein binds molecular oxygen and transports it to cytochrome oxidases that are the final enzyme of the respiratory chain. In this way, bacteria have the ability to survive hypoxic environment (Chien *et al*., 2006). In the case of the introduction of the *vgb* gene into cells of other bacteria, intensification of growth and synthesis of metabolites is very often observed (Ramandeep *et al*., 2001). The *vgb* gene was introduced into *K*. *xylinus* BCRC12334 cells by electroporation using the modified plasmid pBBR122. The constitutive expression of the gene and the presence of active protein in the transformants were confirmed, as was the intensification of the growth of microorganisms, probably due to the increased oxygen uptake. Faster bacterial growth resulted in an increase in the amount of synthesized cellulose, with the yield being about 50% higher in the case of micro‐aerobic cultures than in aerobic conditions (Chien *et al*., 2006).

Another recombinant *K*. *xylinus* BCRC12334 was conceived as a sophisticated strategy to express a protein of interest, supporting its stable expression and allowing its self‐immobilization (in the bacteria) on BNC fibres. As a model enzyme expressed in recombinant *K. xylinus*, d‐Amino oxidase (DAAO; EC1.4.3.3) from *Rhodosporidium toruloides* was used. The constructed *K*. *xylinus* mutant not only successfully produced DAAO activity but was also immobilized by cellulose nanofibres both in stationary and shaken cultures. Although self‐immobilized cells exhibited only 10% of the DAAO activity available in the crude cell extract, they provided several advantages in terms of better thermal stability, stability of operation and easy recovery for repeated use (Setyawati *et al*., 2009).

Fang *et al*. (2015) introduced the Curdlan synthase gene, *crdS*, from *Agrobacterium* sp. ATCC31749, in *K. hansenii* AY201, attempting to combine the BNC and Curdlan synthesis metabolic pathways. A significant difference in the morphology of the bionanocomposite surface was noticed, because the BNC pore structure was covered with Curdlan, which also reduced the water permeability. Although Curdlan's secretion significantly changed the surface morphology of the membranes, it had little impact on the crystalline structure, indicating that Curdlan secretion may be of lower priority than BNC nanofibre production (Fang *et al*., 2015).

Due to the resistance of BNC to *in vivo* degradation, its use in tissue reconstruction is limited. In order to obtain a polysaccharide susceptible to *in vivo* degradation, an attempt was made to introduce into *K*. *xylinus* 10245 cells an operon containing three genes (NAG5, AGM1 and UAP1) responsible for the synthesis of UDP‐*N*‐acetylglucosamine (UDP‐GlcNAc). The operon sequence originated from yeast cells *Candida albicans*. It was assumed that the availability of UDP‐GlcNAc for cellulose synthase would allow the incorporation of GlcNAc into the biopolymer structure. The operon sequence was introduced into *K. xylinus* cells by electroporation using the modified plasmid pBBR122. Transformants able to synthesize modified cellulose containing GlcNAc were obtained, when *N*‐acetylglucosamine was present in the culture medium. Transformants showed a significantly lower BNC yield; however, the polysaccharide obtained was characterized by a high content of GlcNAc, and thus lower degree of crystallinity due to the weak interaction between the fibrils. As part of the research, an analysis of its susceptibility to degradation *in vivo* was also carried out by implanting BNC and modified BNC into mice. It turned out that the polysaccharide containing GlcNAc was completely degraded after only 3 weeks, in contrast to the implant made of native cellulose (Yadav *et al*., 2010).

Modification of BNC structure has mainly been achieved by chemical or physical modifications of the BNC pellicles or changing culturing conditions (Chanliaud and Gidley, 1999; Luo *et al*., 2008; Hu *et al*., 2014). Although genetic modifications of the *Komagataeibacter* strains were aimed mainly at increasing the productivity, the group from Lodz University of Technology recently obtained two disruption mutants producing stiffer membranes with a more‐packed structure and three overexpression mutants producing membranes with a porous structure (Jacek *et al*. 2018) demonstrated that by manipulating genes responsible for motility and cell divisions it is possible to obtain mutants producing BNC membranes with desirable properties. These studies initiate a new promising trend in obtaining BNC membranes with changed structure.

## Perspectives

Bacterial nanocellulose has been gaining significant attention from scientists and engineers in various research fields due to its unique properties. Nevertheless, the high cost of manufacturing and genetic instability of some *Komagataeibacter* strains are major obstacle to its wide application. Genetic engineering of bacterial nanocellulose producers is believed to make this process stable, more efficient and less expensive than today. One of the most impressive examples of genetic engineering is the *K. xylinus* mutant which uses lactose from whey as a carbon source, producing 28‐fold more cellulose from lactose than the wild‐type strain (Battad‐Bernardo *et al*., 2004).

The growing number of available genome sequences from the genus *Komagataeibacter* and genetic tools should allow to better understand the molecular aspects of BNC biosynthesis and rationally engineered mutant strains (Florea, *et al*., 2016a; Pfeffer *et al*., 2016a,2016b; Zhang *et al*., 2017). In recent years, comparative genomic and transcriptomic analyses are gaining attention in the scientific community. Recently, it has been shown that high complexity of regulatory mechanisms, directly or indirectly involved in the cellulose biosynthesis in *Komagataeibacter* strains, could be predicted by comparative genomic analysis (Ryngajłło *et al*., 2018). Transcriptome analysis‐based RNA‐seq is an important technique to identify differentially expressed genes under distinct experimental conditions in bacteria. Compared with genome analyses, transcriptome analyses only evaluate transcribed genes; thereby, they have a smaller research scope, which may lead to more accurate results. Therefore, it is necessary to study the phenotype of *Komagataeibacter* new mutants using RNA‐seq technique because this will reveal new dependencies and predict new genes for further research.

Depending on the application, BNC with varying stiffness, tensile strength, porous or denser structure is required. Modification of BNC structure has mainly been achieved by changing the type of bacterial strains, additional supplements to the medium, and differentiation of their growing conditions. Novel approach to change the properties of bacterial nanocellulose is to genetically modify the bacteria. Moreover, genetic engineering may allow a greater range of biomaterials to be produced, by providing precise control over cellulose synthesis genes and production BNC membranes with desired properties. Thus, the fine‐tuning of the properties of BNC through genetic engineering is being successfully achieved, furthering the potential of BNC for application in biomedicine and other fields. Nevertheless, BNC received from genetically modified strains may face regulatory restrictions in medical and food industry.

## Conflict of interest

None declared.
